# The Political Ontology of Corporate Social Responsibility: Obscuring the Pluriverse in Place

**DOI:** 10.1007/s10551-022-05175-1

**Published:** 2022-08-01

**Authors:** Maria Ehrnström-Fuentes, Steffen Böhm

**Affiliations:** 1 Hanken School of Economics, Kirjastonkatu 16, P.O. Box 287, 65101 Vaasa, Finland; 2 University of Exeter Business School, G11, SERSF, Penryn Campus, Cornwall, TR10 9EZ UK

**Keywords:** Singularization, Sustainability, Pluriverse, Political ontology, Territorial struggles

## Abstract

This article examines corporate social responsibility (CSR) through the lens of political ontology. We contend that CSR is not only a discursive mean of legitimization but an inherently ontological practice through which particular worlds become real. CSR enables the politics of place-making, connecting humans and nonhumans in specific territorial configurations in accordance with corporate needs and interests. We discuss three CSR mechanisms of singularization that create a particular corporate ontology in place: (1) community engagements that form ‘stakeholders’; (2) CSR standards and certifications that produce singular sustainable environments; and (3) CSR reporting that erases ontological conflicts and enables the singularized representation (of the environment and the community) to travel to other locations of the corporate world. We argue that these ontological CSR practices obscure the pluriverse of other world and place-making practices that would create different kinds of sustainabilities based on less extractive and non-corporate ways of being in place.

## Introduction

For centuries, land-based communities have been under threat by settler colonialism, industrialization, extractive industries, and other modern development agendas (Banerjee, 2000; 2018), more recently framed as different forms of extractivism (Chagnon et al., 2022; Ehrnström-Fuentes & Kröger, 2018; Gudynas, 2015; Kruter Flores et al., 2020). In these contexts, where corporate and local worlds meet, conflicts tend to arise. The Environmental Justice Atlas (EJAtlas) lists over 3,600 of such conflicts globally, most of which are struggles over land and territory (Temper et al, 2015). For example, many of such conflicts are over water qualit and landscapes upon which fisherfolk, tourism entrepreneurs or Indigenous populations depend for their own existences, which are, however, affected detrimentally by large-scale industrial, forestry, mining, and windmill facilities (Dunlap & Correa Acre, 2022; Heikkinen et al., 2013; Ehrnström-Fuentes, 2016, 2022b; Joutsenvirta & Vaara, 2015; Littlewood, 2014; Kröger, 2021; O’Faircheallaigh & Ali, 2017). Such struggles tend to emerge in places where trees, mountains and rivers are not consider just as  resources suitable for extractive purposes but sentient ‘earth beings’ (de la Cadena, 2015) that, together with (and inseparable from) people, shape how life is lived in place (Ehrnström-Fuentes, 2022b; Mansilla Quiñones & Melin Pehuen, 2019). These conflicts are ontological in nature, as they go to the core of what Moore (2015) calls the ‘web of life.’In this article, we develop an analytical frame of political ontology (PO) to explain such place-based conflicts in relation to corporate social responsibility (CSR).

PO is a concept developed by Latin American anthropologists (Blaser, 2010; de la Cadena, 2015; Escobar, 2016), providing a political understanding of complex ontological antagonisms between modern and other ways of worlding, which are typical in place-based struggles over land and resources (de la Cadena & Blaser, 2018). Modernity refers explicitly to a particular ontology (or myth) that singularizes and occludes the multiplicity of other worlds (the pluriverse) through processes of colonial domination, based on three assumptions: (1) subjects are seen as detached from objects, and people as detached from place and nature; (2) time is constructed in linear ways, rendering some people and cultures as more advanced (or ‘civilized’) than others; (3) the power differential between ‘moderns’ and other ways of being, knowing, and sensing legitimizes the colonization (and destruction) of other worlds (the colonial difference).

Modernity should therefore not only be understood as a social process of global expansion based on ideas of European origin (e.g., Giddens, 1991; Habermas, 1996), nor is it enough to simply consider the multiple or alternative modernities that are dependent on locally grounded cultural and epistemological interpretations of ‘the world’ (in singular) (e.g., Eisenstadt, 2002; Gaonkar, 2011). Instead, PO examines how modernity, as an ontological construct and very particular kind of world, disregards other forms of performing the world by classifying them—not always consciously though—as ‘ignorant,’ ‘irrelevant,’ or simply not ‘real’ enough to count as important. Thus, PO’s analytical focus lies on place-based communities defending ‘alternatives to modernity’ (Escobar, 2008) and whose relations to nonhumans do not necessarily follow the modern rationale of dualism and separation (de la Cadena & Blaser, 2018; Escobar, 2020).

In this article, we argue that CSR plays a crucial role in the ontological politics of place-making. CSR has concrete impacts on human–nonhuman relations as it transforms the way in which people live and relate to place in their everyday lives. A political ontology view of CSR pays close attention to place, understanding how it is assembled through historically sedimented practices and, influenced by the modern ontology, naturalizes certain human and nonhuman entanglements at the expense of others (Ehrnström-Fuentes, 2016, 2022a; Law & Lien, 2018). However, given that places, just as bodies (Mol, 2002), always exist in multiple ways, CSR’s shaping of place obscures the pluriverse, that is, the multiple other ways of being in and relating to humans and nonhumans in place (de la Cadena & Blaser, 2018).

Business ethics scholars have long developed critical perspectives of CSR, understanding its political and power dimensions (Banerjee, 2018, 2021; Böhm et al., 2008; Whelan, 2012; Fleming & Jones, 2012; Scherer & Palazzo, 2011; Vallentin & Murillo, 2012). While territory and land have been identified as vital by some CSR scholars (Banerjee, 2000; Ehrnström-Fuentes, 2016, 2022b), there has been a general lack of consideration of the struggles over place (Ehrnström-Fuentes, 2022a). This is particularly important when considering the way Indigenous people are being treated in the encounter with the (modern) corporate world, but also extends to other communities (e.g., peasants, artisanal fisherfolk, small-scale tourism communities). The life and subsistence practices of these local groups are deeply rooted in the land on which they live (Ehrnström-Fuentes, 2016, 2022a, 2022b), and their ways of practicing responsibility together with nonhumans in place create wholly different sustainabilities to those of corporations (Jääskeläinen, 2020; Virtanen et al., 2020).

The purpose of this article is to develop a PO framework for understanding CSR, which, we argue, has become one of the most important corporate means to act both responsibly and sustainably in place. However, as we will show, CSR investment reports, environmental labels and sustainability indicators reproduce modern ontological assumptions that legitimize corporate extractive land-use practices, occluding other ways of being in place and interrupting the possibilities of imagining alternative, place-based sustainability practices. A PO approach affords us to understand CSR as an ontological practice that creates specific places and worlds, leading to place-based conflicts over land and resources. As climate change and other environmental crises are predicted to intensify during the *twenty-first* Century (Jiricka-Pürrer & Wachter, 2019), such conflicts are likely to increase, making it more urgent than ever to develop ways of seeing other ontologies, alternatives, and non-corporate ways of building sustainable futures in place.

## The Politics of CSR

It has long been understood that CSR is closely entangled with political processes (Frynas & Stephens, 2015; Sheehy, 2015; Whelan, 2012). There are at least four main approaches to understanding the politics of CSR. First, the processual and micro-foundational perspective of CSR (Gond et al., 2017) highlights the political dynamics of CSR processes within corporations (Gond & Moser, 2021). Here, the focus is on firm–internal processes and struggles that involve political discourses, ideologies, identities, and issue-selling (Chin et al., 2013; Gupta et al., 2017; Kourula & Delalieux, 2016a, 2016b; Wickert & De Bakker, 2018). This literature reminds us that within organizations there is always a plurality of logics as well as moral and political positions at play, producing different internal and external outcomes (Demers & Gond, 2020). This micro-politics also involves human and nonhuman actors that materialize CSR (Gond and Nyberg, 2020; Jensen & Sandström, 2020).

Second, CSR has been analyzed through the lens of political systems (Djelic & Etchanchu, 2017). Politics in this context refers to firms’ relations to governments (Mäkinen & Kasanen, 2016; Zueva & Fairbass, 2021) and political institutions (Mäkinen & Kourula, 2012) —what is sometimes called ‘corporate political activity’ (Fooks et al., 2013; Nyberg, 2021)—as well as their often conflictual dealings with a range of civil society groups, including non-governmental organizations (NGOs) and social movements (Sorsa & Fougère, 2020). Sheehy (2015) sees CSR as part of a general shift toward corporate self-regulation, which Scherer and Palazzo—through their ‘political CSR’ perspective (Palazzo & Scherer, 2006; Scherer et al., 2016)—translate into a normative call for a politicized role of the firm (Fairbrass and Zueva-Owens, 2012) that is not reliant on governments. Instead, corporations are encouraged to deliberate directly with civil society actors to regulate aspects of their shared concerns, managing the provision of public ‘goods’ and restricting public ‘bads’ (Palazzo & Scherer, 2006; Scherer et al., 2016). Several authors (Moog et al., 2015; Djelic & Etchancu, 2017; Rhodes & Fleming, 2020) critique the political CSR framework for being embedded in a neoliberal model that assumes that the rise of corporate self-regulation and the assumed decline of the state are universal, unquestionable developments. Yet, as many scholars point out (Ehrnström-Fuentes & Kröger, 2018; Whelan, 2017; Zueva & Fairbass, 2021), the state continues to be important, as has been plain to see during the COVID-19 pandemic. In addition, as Whelan (2017, 2019) rightly argues that, beyond firm–civil society and firm–government relations, corporations’ daily ‘bread and butter’ of creating and selling products and devices should also be considered political. For example, during the recent war in Ukraine, many companies had to make a decision of whether to withdraw their operations from Russia or not, which was seen by many governments and civil society actors as deeply political. As we will argue, however, this perspective typically excludes considerations of how corporations transform ‘whole webs of existences, practices, species, cultures, livelihoods, sustenance possibilities, and so forth’ (Kröger, 2021, p. 3).

Third, scholars have examined the politics of CSR as legitimation discourse and form of propaganda (Hanlon & Fleming, 2009), supporting the interests of (extractive) corporations while obscuring colonial processes of dispossession in the Global South (Banerjee, 2000, 2018; Alcadipani & de Oliveira Medeiros 2020; Blowfield & Frynas, 2005; Özkazanç-Pan, 2018). These scholars see CSR as a tool for greenwashing (Lee et al., 2018; Mahoney et al., 2013; Siano et al., 2017), hiding colonial violence (Banerjee, 2000, 2008, 2018, 2021; Ehrnström-Fuentes, 2016) while disregarding other logics, perspectives and lived realities in the Global South (Alcadipani & de Oliveira Medeiros 2020; Maher, Monciardini, et al., 2021). These are meaningful and important critiques of CSR and its impacts on marginalized and often land-based communities. Yet, most of these studies remain centered on the institutional politics within a political economy domain rather than interrogating the material politics of place and the ontological multiplicity that comes with radically different ways of performing the world (Ehrnström-Fuentes, 2022a, 2022b). There have been some recent calls for ‘a more Earth-centric perspective in organization and management studies’ (Banerjee & Arjaliès, 2021, p. 3, see also Ergene et al., 2021), suggesting that scholars are becoming increasingly interested in radically different materialities and ways of performing the world. Yet, they remain sparse.

Fourth, scholars have considered the material dimensions of CSR and the dynamics of human–nonhuman assemblages, focusing on CSR reporting devices (Gond & Nyberg, 2017). Yet, this seldom involves a consideration of the physical dimensions of place (Jensen and Sandström, 2019) as well as the experiences of communities directly affected by CSR practices (Maher, Monciardini, et al., 2021). From a community perspective, what is considered ‘sustainable’ and ‘responsible’ depends on the ontological assumptions that underpin how people in the community relate to their own lived-in world. This involves human–human but also human–nonhuman relations, such as people’s engagements with plants, animals, mountains, and waterways (Virtanen et al., 2020). These human–nonhuman ontologies are not static but are continuously evolving (Law, 2004; Law & Lien, 2018; Mol, 2002). Thus, when communities engage with the materialities of CSR, these entanglements also shape their own ontologies, or their ways of worlding their world in place.

In this article, we argue that such ontological analysis of what CSR does to places (and worlds) is largely missing in the literature. We maintain that corporate engagements in CSR shape places in particular ways, which, in turn, has concrete impacts on the community’s web of life as well as the different life-sustaining relations in place (Ehrnström-Fuentes, 2022b) and the materialities (e.g., housing and education facilities) that emerge from such engagements. Thus, the politics of CSR is not just expressed as abstract representations produced by institutional or discursive struggles (Gond & Nyberg, 2017; Joutsenvirta & Vaara, 2015) but as ontological struggles over what is un/real and what can and could exist in place. Next, we present the political ontology frame that can help us build a robust theoretical frame of how CSR contributes to the ontological politics of place-making.

## Political Ontology

The idea of the existence of multiple ontologies beyond modernity initially emerged out of the work of anthropologists that explored the metaphysical constitutions of worlds by mapping different Indigenous ontologies and their specificities in relation to modernity (e.g., Descola, 2005; Viveiros Castro, 1998). Drawing on Science and Technology Studies (STS) and in close dialogue with Indigenous interlocutors in Latin America, PO adds to this debate by showing that such ontologies do not exist as pre-given, clearly distinguishable and separate ‘worlds.’ Instead, they are brought into being through multiple world-making practices (or ‘ways of worlding’) that interact, interfere, and mingle with each other (Blaser, 2013a; de La Cadena, 2015; Escobar, 2020). This implies that, for PO, there is no external reality ‘out there’ and hence no separation between the observer and the observed. Instead, how people relate to other beings and things through their own ways of worlding will shape the materialities brought into being (Law, 2004; Mol, 2002). Thus, ontologies are political, because how we humans relate to the world beyond ourselves matters in real material terms for the world(s) we bring into being (de la Cadena & Blaser, 2018).

In PO, the term ‘ontology’ (or ‘world’—we use these words interchangeably) is understood through three interlinked registers. First, it refers to the implicit and explicit assumptions that a social group makes about the kinds of ‘things’ that exist in the world and the conditions of their existence (Blaser, 2010). The proposition of PO suggests that differently constituted ontologies (e.g., totemism, animism, naturalism, analogism) (see Descola, 2005) do not produce different cultural and epistemological perspectives about one single nature ‘out there’ (which would be aligned with ideas on multiculturalism, or pluralism). Rather, different ontologies produce different realities, materialities, natures, and worlds. Hence, these worlds are composed of a ‘pluriverse’ of many different overlapping worlds with no clear boundaries or overarching principles that would turn this multiplicity into a singular world (Blaser, 2013a; Escobar, 2020). It is the dominance of the modern ontology and its ontological assumptions that convert this multiplicity into representations of a singular world ‘out there,’ occluding the diverging world-making practices of the pluriverse. The proposition of PO is to examine the power relations that enable this singularization of worlds by tracing emerging dynamics of conflict and appropriation (Blaser, 2009), paying attention to the researcher’s own participation in the occlusion of the pluriverse (Blaser, 2010, 2013b).

Second, in line with STS scholarship, PO understands ontologies to perform themselves into being (Blaser, 2009). This means that ontologies are not schemes of classification but enacted or performed in practice (Law & Lien, 2018; Mol, 2002), which involves humans–nonhumans entanglements (see Latour, 1991, 2005; Law, 2004; Mol, 2002). Specific entities acquire their socio-material attributes based on their relations to others (Law, 2004). For example, objects such as ‘water’ and ‘land’ always exist as multiples and in-between persons, things, institutions, and other-than-human beings (Stensrud, 2016). The point is not to examine practices ‘as they are’ but to understand how they come into being and perform particular kinds of realities, worlds, or natures (Stensrud, 2016). The crafting of realities and statements about the world and its materialities are produced together with particular scientific practices that include specific instrumental, technical, and human configurations (Latour, 2005). Crafting, for example, does not only imply human agency and skill. Rather, ‘people, machines, traces, resources of all kinds—and [in some contexts] spirits or angels or muses—are all involved in the process of crafting’ (Law, 2004, p. 55). This suggests that the world is multiple, produced in diverse and contested social and material relations (Law, 2004).

Mol’s (2002) ethnographic exploration of atherosclerosis disease provides a clear example of how this multiplicity is enacted in practice. Drawing on experiences of patients, radiologists, laboratory technicians and doctors, Mol finds that the same disease assumes different shapes depending on how it is enacted through different assemblages made of people, medicines, technologies, medical records, surgery instruments, feelings, and waiting rooms. Each assemblage enacts a different reality of the same body. This multiplicity is rendered singular through a series of politico-managerial procedures that selectively discard some manifestation in favor of others, yet, at the level of practice, the body always exists as multiple (Mol, 2002). Considering this ontological multiplicity avoids reducing difference to cultural or epistemological perspectives about ‘the world’ (or the body, in the case of Mol’s work). Instead, it enables us to map multiple ontologies enacted through different sets of situated practices. We argue that in the case of CSR, too, different practices contribute to creating a different world in the same place.

Third, ontologies are manifested through stories (Blaser, 2009, 2010). These stories do not ‘float’ above some ultimate reality ‘out there.’ Rather, different stories produce different ontologies (Blaser, 2014). That is, stories perform worlds, contributing to the creation of the reality they narrate (Blaser, 2010), and thus these stories cannot be fully grasped without reference to their world-making effects (Blaser, 2014). What this means is that some stories can be wrong but not because of their ‘lack of coincidence with an external or ultimate reality, but in the sense that they perform wrong, they “world” worlds we do not want to live in or with’ (Blaser, 2014, p. 55). Thus, in PO, storytelling is not a descriptive account of an ultimate reality out there, but a way of worlding with political implications because how the world is performed through storytelling makes ‘some realities realer, others less so’ (Law, 2004, p. 67). Some realities become more (corpo)real (‘material’) than others because of how the imaginations they produce are translated and entangled in communicative threads of reality-making (ibid.).

This understanding of ontology as performed through stories is important when examining CSR, which includes practices that are not only situated in specific places but, as we will discuss further below, involves performing certain kinds of worlds and imaginations that travel across multiple stakeholder networks of the corporate world (e.g., in the format of global standards, sustainability indicators, and CSR reporting).

## The Political Dimension of PO

While STS scholars stress the ontological politics of practices that entangle people, natures, technologies, and things to produce ‘ontologically multiple fragments of being and of the world’ (Eitel & Meurer, 2021, p. 6), PO scholars have taken this proposition further by connecting ontological politics with the power asymmetries that exist between modern and nonmodern worlds. Situated in contexts marked by colonial experiences and power imbalances, PO incorporates insights from decolonial and subaltern studies to examine how the asymmetries between the modern and the relational (‘nonmodern’) produce ontological conflicts about ‘the things at stake’ in land-based struggles where different worlds overlap and mingle (Blaser, 2013a, 2013b). Thus, PO scholars’ primary interest lies in critically examining how the dominance of modernity over other ontologies naturalizes the existence of one singular nature, environment, or world ‘out there,’ thereby depoliticizing the multiplicity inherent to human–nonhuman relations. Consequently, PO’s analytical concern is to make visible the often ignored ontological politics that hide behind the often shining surface of modernity marked by industrial development, consumption, and other images of economic progress.

In this context, ‘modernity’ is understood as an ontology (or myth) sustained through stories that have performative effects on the world. Modernity is thereby not considered a historical period but specifically refers to the modern ontological arrangement, or ‘the modern myth.’ This myth reproduces itself through three constitutive ontological assumptions: ‘the great divide between nature and culture (or society), the colonial difference between moderns and nonmoderns, and a unidirectional linear temporality that flows from past to future’ (Blaser, 2010, p. 5).

The distinction between nature and culture creates practices that regard the individual as independent subject as detached from objects and people as detached from place and nature (Escobar, 2020). Hence, the ‘universal sciences’ generate generalizable facts and observations about the world ‘out there.’ Law and Lien (2018, p. 151) point out how the origin of a separate ‘Nature’ lies in the monotheistic religious tradition of Christianity with the idea of a single deity that ‘imposed an order of formless matter to create a single cosmos with a particular nature.’ With the Enlightenment, the idea of a single and separate nature was carried over to the natural sciences, where the role of science became the discovery of the mechanisms through which ‘Nature’ can be controlled and managed (Ibid.). Through scientific papers, this idea of nature as a separate, discoverable unit independent of context and observers is institutionalized (Ibid).

This includes an unilinear conception of time, which constructs an imagination of the future that rest on the assumption that all communities across the world will follow the path of ‘advanced’ and ‘civilized’ societies toward increased industrialization, progress, material development and globalization (Escobar, 2008). The power asymmetries of the colonial difference between the ‘superior’ (or ‘advanced’) modern and the ‘inferior’ (‘primitive’) nonmodern is what enables the universalization of modern categories of thought.

Ontological conflicts arise when the (modern) abstracted and the (relational) contextual way of relating to ‘the things at stake’ do not converge (Blaser, 2013a, 2013b). Hence, in PO, the political moment is always conceptualized in relation to the dominance of the ontological assumptions derived from the modern myth. The struggle over ‘facts’ and ‘realities’ (enacted through ontological politics) is thereby either manifested in situations where modern assumptions achieve interpretational dominance (streamlining, subordinating, eliminating multiplicity) over other worlds (Blaser, 2010; Eitel & Meurer, 2021), or when ontological conflicts make visible the diverse ways of worlding of the pluriverse, challenging the universality proclaimed by the modern myth (Blaser, 2010, 2013b).

Escobar (2016) uses the concept of ‘the One-World World’ (OWW), developed by Law (2015), to show how modern ontology acts as a world ‘that has arrogated for itself the right to be “the” world, subjecting all other worlds to its own terms or, worse, to non-existence; this is a World where only a world fits’ (Escobar, 2016, p. 15). He explains how the operations of the OWW enable the conversion of everything that exists ‘into “nature” and “nature” into “resources”; the effacing of the life-enabling materiality of the entire domains of the inorganic and the nonhuman, and its treatment as “objects” to be had, destroyed, or extracted; […] to “world markets” for profit’ (2016, p. 18). This ‘ontological capture and reconversion by capital and the State’ (2016, p. 19) is marked by multiple forms of organized violence (Böhm & Pascucci, 2020), denying other worlds the possibility of existing on their own terms. Here, we should be careful, however, not to singularize the OWW either, as Law and Lien (2018) warn us. Their account of how  salmon farming in Norway constructs particular natures shows that the pluriverse extends to ‘modern’ societies too. That is, there is no singular nature within the modern either.

What is, then, the role of the researcher in this context of multiple worlds, natures and realities that are dependent on the practices that create them? If the world exists as multiple, then the kinds of stories we, as researchers, tell matter in real, material terms. That is, the stories we tell about CSR and other business approaches contribute to performing certain kinds of worlds into being, while obscuring others. Our own blindness toward the singularities and hierarchies built into the modern ontological assumptions is part of the politics of PO, as many researchers, often subconsciously, reproduce singular representations that obscure the multiplicity of practices and human–nonhuman relations that exist in place.

To tackle the blind spots of our own ontological horizon requires that we engage in the stories of those whose worlds are endangered by the practices of extractivism and CSR, thereby disrupting the OWW and making visible the pluriverse in our academic writings. Blaser (2010) calls this methodology ‘border dialogue,’ which helps researchers to tell stories of the present through an explicitly moral stance, not taking the modern myth as the ultimate and only ontological condition. The researcher is thus committed to articulating different worlds in symmetrical terms, instead of reproducing knowledge hierarchies created by the OWW.

## PO’s Missing Focus on Corporations

To date, most PO research has focused on participatory environmental governance, involving states, NGOs, and local populations in the negotiations of sustainable hunting (Blaser, 2009), water treatment (Stensrud, 2016), marine life (Schiefer, 2021), and reindeer herding (Johnsen, 2017). These studies all conclude that the ontological hierarchies between modern and other ways of worlding persistently weakens local populations and enforces external and government positions (Eitel & Meurer, 2021).

Blaser (2013a, p. 18) provides a detailed account of how ‘the various agents of modernity (e.g., governments, corporations, environmentalists)’ through participatory governance schemes justify the destruction of the pluriverse by either urging for the conversion of forests and mountains into commodities that fuel ‘economic growth,’ hence ‘progressing’ the ‘greater good of society,’ or, indeed, protecting them as ‘delicate ecosystems’ (ibid., p. 18). In these debates, those nonhuman relatives, spirits, and ancestors (or ‘earth beings,’ see de la Cadena, 2015) that do not fit the purview of science are considered as human fabrications enacted from within the domain of culture and, hence, are not seen as ‘real’ or ‘rational.’ That is, those who only have culture can claim ‘their right to keep their identities, their cultures, and their beliefs’ (ibid., p. 18) but are not taken seriously when they speak about nature on their own terms. Thus, although national and international frameworks increasingly recognize the rights of Indigenous people to be consulted (Jääskeläinen, 2020; O’Faircheallaigh & Ali, 2017, Virtanen et al., 2020), they can often only voice their concerns based on what is considered reasonable (or real) within modern categories of thought, which are based on separations between humans and nonhumans as well as cultures and natures (Escobar, 2020).

The role of corporations in the encounters between local and corporate worlds has been scarcely explored in PO (Ehrnström-Fuentes, 2022a). Instead, corporations are referred to, in rather abstract terms, as ‘capital’ (Escobar, 2008, p. 69–110), or as background entities that, through state intermediaries, provoke conflicts with local ways of relating to the nonhuman world (Jääskeläinen, 2020; Sepúlveda, 2016; Stensrud, 2016). Yet, in many places the state is either absent or invisible, often leaving corporations to become state-like entities (Maher et al., 2019; Maher, Huenteao, et al., 2021) with direct influence in the everyday lives of the local population (Acosta et al. 2019).

Sepúlveda’s (2016) study of a serious pollution incident near the Southern Chilean town of Valdivia in 2004–2005 points at the central role of corporations in local struggles over place. Her examination of the mass death of a rare species of swans next to an Arauco pulp mill shows how such a visible disaster can lead to local mobilizations that cause an ontological fracture in how people relate to the more-than-human world. The death of the swans unsettled modern relations to the environment (in which nature is viewed just as resource) and made previously hidden humannonhuman relations surface, creating demands for change.

This and many other such cases confirm that there can be no doubt that the OWW is marked by multiple forms of organized violence, mostly initiated by states and corporations (Böhm & Pascucci, 2020; Costas & Grey, 2019). Whereas in the past nations such as England and Spain colonized foreign land and their people by sheer military force exercised by state armies, corporate trade and other forms of ‘soft power’ have always been part of the violent politics of the OWW. Today, this double force of violence persists. For example, many corporations maintain armed ‘security’ forces and/or work closely with states’ police and military personnel to ‘secure’ their operations (Böhm & Pascucci, 2020; Schmalz et al., 2022). The rapidly increasing number of assassinations and criminal prosecutions of local environmental defenders (Dunlap, 2021; Hadad et al. 2021; Tran et al. 2020), also in territories occupied by ‘green’ and ‘sustainable’ industries (Dunlap & Correa Acre, 2021; Schmalz et al., 2022),  are bleak examples of this brute OWW violence.

Yet, we maintain that the ‘soft power’ of CSR is part of the same violent OWW regime, which is often forgotten or ignored by CSR scholars (Banerjee, 2008). In fact, after the 2004–2005 crisis in Valdivia, many Chilean companies started to invest in CSR activities (Sepúlveda, 2016). This calls for further analysis of the role of CSR in the ontological politics of place-making. Sepúlveda’s case study suggests that corporation may very well use community projects and environmental programs labeled as CSR to manage the risk of local (and ontological) conflicts that could endanger the presence of the corporation in place. In this way, the ‘soft power’ that CSR appears to be, becomes part of the violent OWW regime. Today, the Chilean forestry company Arauco paints itself in ‘carbon neutral’ and ‘responsible’ colors, yet, many Indigenous communities still mobilize to resist their forestry practices, as they are seen as a violent colonization of the Mapuche’s world. We argue that CSR is the logical continuation of centuries of violence and colonization practiced by the OWW. In the remainder of this article, we will examine in more detail how specific CSR practices and mechanisms produce and reproduce the OWW. Corporations play a vital yet singular role in this OWW, turning local communities and their natural environment in stakeholders and reportable data, standards, and categories, thereby obscuring the pluriverse of other worlds.

Our own interest in examining CSR as an expression of PO that reduces the multiplicity of the pluriverse to a singular OWW starts from engagements in border dialogues with local interlocutors whose lives (and worlds) are endangered by corporate practices of extraction. Through many years of engagements with land-based and alternative communities, we have engaged in countless conversations with people who do not want to take part in the ‘development’ offered by corporations. It is in this problem space ‘in-between worlds’ where our enquiry into CSR started, which involves investigating how also our own taken-for-grated (modern) assumptions about ‘the world’ contribute to legitimize certain CSR practices and reproduce the dominance of the OWW at the expense of the territorially embedded pluriverse.

## The Political Ontology of CSR: Three Mechanisms of Singularization

As we have argued above, CSR needs to be understood as part of the OWW that has been historically shaped by societal, cultural, technological, and economic practices and philosophies of European origin. Through processes of colonization and globalization, CSR has spread to novel places, where, blended with local histories, it has shaped communities’ realities in various ways. In this section, we identify three mechanisms through which CSR practices have (historically) shaped the ontological politics of place-making, strengthening the OWW at the expense of other worlds. These mechanisms include processes that seek to: (1) create stakeholders with shared values (ontological capture of place); (2) settle the stakes in place through standardized certifications (erase ontological conflicts in place); and (3) re-present local realities through stories, images, and indicators that, just like maps, can travel through space (ontological singularizations through space). We do not claim that these are the only mechanisms through which corporations’ CSR activities contribute to the ontological politics of place-making. Nevertheless, our reading of the literature and our own dialogues with local interlocutors and research experiences over the past two decades have led us to identifying these three mechanisms as significant.

## CSR as Community Engagements—Creating *StakeHolders* in Place

This mechanism focuses on how seemingly independent actors in place are brought into the corporate network as ‘stakeholders,’ supportive of the corporate *stakes* in place. This approach goes back to the England of the industrial revolution, which gave rise to philanthropy and the idea that wealth accumulated by industrial owners should also contribute to improving society (Latapí Agudelo et al., 2019). Through processes of globalization—supported by the idea that the ‘advanced’ nations in the global North have the moral obligation to bring progress and development to ‘impoverished’ communities in the global South (Escobar, 2008) —these practices have spread across the world. Analyzing a case from Colombia, Acosta et al. (2019) show how local realities are constructed through inherited realities from the past:

CSR activities in Colombia are rooted in the Catholic tradition and built on a long history of corporate philanthropy. SugarCo reflects this history, by supporting many aspects of life outside the factory […] On the industrial premises, there is […] a church, a theatre, a swimming pool, and a store where employees and villagers can buy appliances and groceries on Saturdays. SugarCo also provided transportation for employees and the local community. As evidence of the difficulty of demarcating the professional and private spheres, many employees and the community tend to consider the company a ‘family’ […] The company also provides healthcare services. (p. 1117–1118)

This excerpt shows how the lived reality in place is dependent on the presence of the corporation and its CSR investments. Through their entanglements with company-sustained buildings and services, the private sphere of community members becomes integrated with the corporate sphere. These CSR entanglements convert community members into corporate stakeholders, whose stakes are defined based on what is deemed desirable from within the OWW. As the corporate world creates concrete materialities and core services for the community, CSR generates an assemblage of people, technologies, things, and natures that change how locals live their lives. Given that CSR is only the latest of such place-making in a long line of colonizing practices, we can talk about the extension of an ‘ontological occupation’ that has shaped subjectivities in ways that make radically different futures unthinkable (Escobar, 2020).

Acosta et al. (2019) also describe how these CSR practices have changed as a result of demands coming from MNCs, requiring a more formalized approach (Waddock, 2008). As a result, the company moved ‘away from a paternalistic relation of dependence by surrounding communities’ to, instead, ‘empower the local communities and help them express and review their needs in a more structured way’ (Acosta et al., 2019, p. 1120). This ‘participatory CSR’ is supposed to empower communities, yet, as it is company-sponsored, cannot question the corporate presence and its (adverse) impacts on the community (Infante, 2020). Instead of helping communities sustain their own lives, these dialogues change how people relate to themselves and their land on terms defined by the corporations. This often creates deep divisions within the community, as some choose to engage with such CSR dialogues and others prefer not to participate at all (Infante, 2020).

Blaser (2010) explains how this type of ‘participation’ extends the network of powerful interest groups (e.g., corporations) to include local communities in ways that does not threaten the OWW but strengthens it. By teaching communities how to represent themselves within the OWW logic, CSR consultants make sure that their inclusion happens in nondisruptive ways. When their own ways of framing their world is considered unrealistic (because it goes against the OWW logic), they are trained to represent themselves ‘accurately’ so that they can contribute to their own development (as defined by the OWW). Local groups are also carefully selected (radical groups are left outside participation), to make sure that the claims raised during the participatory process are not disruptive (Blaser, 2010).

This more ‘inclusive’ approach—also called ‘stakeholder management’ (Freeman, 2001) and ‘co-creation of shared values’ (Porter & Kramer, 2011)—not only stresses the collaborative relations that corporations can have with local communities (Bowen et al., 2010; Dougherty & Olsen, 2014; Heikkinen et al., 2013) but is also said to improve firms’ financial performance and secure their ‘social license to operate’ (Prno & Slocombe, 2012). In this way, corporations can combine—at least in theory—societal objectives with the competitive needs of the market (Mena & Palazzo, 2012; Scherer et al., 2016). While through such ‘participation’ the OWW is never challenged as such, an illusion is created that local ‘cultures’ have been incorporated into firms’ CSR efforts.

From the community’s point of view, a paradox is hence created. The same corporation that pollutes and damages their place also provides the material infrastructure that sustains life in the community (e.g., work, education, health). This makes it very difficult or even “irresponsible” for community members to demand that the corporation should leave (Infante, 2020). As a result, communities are subjected to subtle processes of ongoing colonization, as Infante notes:

The wicked magic […] is that it operates on an unconscious level. Consciously, it is evident that extractivist companies bring impoverishment, pollution, family breakups, loss of values, deterioration of the territory […and] the State plays the role of an ally for the companies, protecting transnational businesses and systematically ignoring the communities. […] [W]e know what we are capable of, we have lived for centuries without mega-companies that come to save us, and we have enough history and knowledge to be self-sufficient in our territories. However, when we give in to the manoeuvres of the companies, we find ourselves suddenly accepting the multicourt, the ambulance, scholarships, computers, the mega highway, new buildings for the community association. Even when we intuitively know that this is just a small compensation for our way of life and our autonomy… (2012, p. 3, authors’ translation)

It is important to also note that, often, locals cannot disregard offers to participate in CSR dialogues, as many communities face ‘structural poverty,’ including the absence of basic services (e.g., schools and hospitals), pushing them to accept whatever they are offered (Blaser, 2010; Infante, 2020). Such desperate condition disciplines locals into desired forms of conduct that further strengthens the OWW (Blaser, 2010, p. 185).

## CSR standards and Certifications: Creating Singular Sustainable Environments

Here, we focus on the practices of standardizations, certifications and other novel ‘CSR infrastructures’ (Waddock, 2008) that stabilize particular representations of CSR as ‘sustainable.’ Standardized codes of conduct and social and environmental certifications—for example, Forestry Stewardship Council (FSC), Roundtable on Sustainable Palm Oil (RSPO), Fairtrade—have become central governance tools. These novel devices, which are often determined and monitored by multi-stakeholder governance systems (Levy et al., 2016; Moog et al., 2015), expand corporations’ responsibilities toward a wide variety of stakeholders and natures (Waddock, 2008). Our argument is that these CSR standards and certifications occlude ontological multiplicity of diverging human–nonhuman relations by creating singular representations of the environment in place.

As discussed above, from within a nonmodern, relational ontology, entities such as ‘the environment’ or ‘nature’ do not exist on their own. What exist as entities always come into being through the situated relations and practices that sustain them, which means that the entities that emerge from these relations will depend on how people connect with the nonhuman world. From the logic of the modern ontology, however, nature (or ‘the environment’) is assumed to exist as a singular entity separated from culture (Law & Lien, 2018). Through modern articulations, such entities acquire the status of being ‘representative’ of reality ‘out there’ (Blaser, 2010, p. 153). Hence, in the modern way of representing nature, the diverging ways of relating to nonhumans become overridden by the concern of streamlining, subordinating, and eliminating difference (Blaser, 2010). In such context, difference can only be expressed as cultural or epistemological perspectives of the real world ‘out there,’ where modern perspectives have interpretational dominance over others. This is precisely the task of CSR standards and certifications. By streamlining the human–nonhuman entanglements into singular representations of ‘the environment,’ CSR articulates equivalences across diverse landscapes of human–nonhuman entanglements. This in turn obscures the ontological multiplicity in place.

This is a different understanding to Gond and Nyberg’s (2017) conceptualization of what they call ‘CSR agencements,’ which refer to the human–nonhuman assemblages that have the capacity to act and produce material effects on the world. They argue that the exclusion of some aspects of their materialization produce overflows of negative externalities, such as pollution or climate change effects, which in turn creates opportunities for political interventions and mobilizations (Gond & Nyberg, 2017). However, the performativity of these ‘agencements’ do not just bring novel, political CSR representations into being, as they hope. Based on our PO conception, we argue that these CSR agencements naturalize corporate–nonhuman entanglements as a singular environment in place, while obscuring other human–nonhuman entanglements that make (or could make) a place into something else. In other words, CSR certification and standardization processes not only erase ontological differences, but they also occlude the pluriverse of multiple other ways of performing the world.

The possibility for local populations to influence the certification procedures is very limited, as they are settled in multi-stakeholder networks to which local communities are not granted access (Banerjee, 2018, 2021). Although public hearings or stakeholder dialogues (Ehrnström-Fuentes and Kröger, 2017) do often take place, the legitimacy of local communities’ demands still depends on whether they can be scientifically evidenced by ‘expert’ knowledges that fit the modern parameters of what is considered ‘reasonable’ (Blaser, 2013b). Hence, by applying modern ‘reasonable’ assumptions about what can exist in place, these systems of global multi-stakeholder governance streamline, subordinate and eliminate local ways of worlding by converting complex relations among humans and nonhumans into abstract environmental representations that can be monitored and controlled in a standardized way.

The standards held by the above-mentioned Arauco’s tree plantations serve as an example of how this ontological multiplicity is erased. The Chilean MNC Arauco has followed the FSC standard and recently announced that it is ‘the first company in the world’ to be certified as ‘carbon neutral’ based on two international standards (Deloitte’s Carbon Neutrality Standard, and Science Based Targets initiative) (Arauco, 2020). Yet, when Indigenous Mapuche communities express their concerns about how the tree plantations have affected their world they do not mention carbon dioxide nor the criteria for sustainability set by the FSC. Instead, their Indigenous stories speak of how the arrival of plantations has seriously affected the balance in their *mapu* (the land to which the Mapuche belong). As expressed by a Mapuche woman:

When the *ngen* leaves, your spirit dies, you no longer have your [nonhuman] brothers, you become *kuñifall* (orphan), because the *ngen* left and is no longer there. The *ngen* got angry with you because you didn’t defend it, because you let it go... In the Mapuche world, nothing is insignificant, everything has transcendence, everything exists in symbiosis, and if the *ngen* goes away it creates an imbalance. … in the Mapuche world, we are a totality and one without the other does not exist. If we continue to allow the foresters to continue to be our neighbours knocking on the door of our house, what we are doing is that we are disappearing, we are committing suicide. (cited in González Correa, 2019, p. 87, authors’ translation)

Through the representations of standards and certifications, the singular corporate approach to ‘sustainability’ is naturalized, obscuring the pluriverse of complex human–nonhuman entanglements. These complex life-sustaining relations in place cannot be understood from a modern perspective of what nature ‘is.’ The Mapuche *ngen*, which inhabit native forests as a life force, connecting all beings in place, disappear with the arrival of monocultural tree landscapes, certified as ‘sustainable’ and ‘carbon neutral.’ We argue that the systems of governance that mobilize and control how ‘the environment’ is represented through such CSR standards and certification schemes do not only restrict ‘the plurality of interpretations’ (Gond & Nyberg, 2017); they effectively obscure the complex human–nonhuman relations that sustain the pluriverse and place-based practices that run counter to the kind of industrial practices and territorial occupations that these certifications represent. Thus, these CSR representations hide the ontological conflict that exists between corporate (industrial, extractive, large scale) practices, on the one hand, and local, land-based community practices, on the other.

## CSR Reporting: Singularized Representations that Travel Across Space

The above outlined CSR mechanisms of creating stakes, stakeholders, and the environment in place feed into the reporting practices of the corporation, making these ‘settled’ representations of the local world, which then travel to other parts of the corporate network. CSR reporting practices can only be performed after the ontological struggles over the stakes, stakeholders and the environment in place have been settled, as otherwise the corporation risks being exposed to accusations of greenwashing, because of visible conflicts that manifest themselves in other non-corporate articulations of local worlds. Thus, it is in the interest of corporations to distance themselves from the more conflictual encounters with locals. This might explain, for example, why the more violent features of the forestry conflicts in Mapuche territories in Chile are contained by state agencies (e.g., police and military forces), while the corporations dedicate a considerable number of resources to their community engagement and CSR practices to co-create ‘shared values’ (Ehrnström-Fuentes and Kröger, 2017).

Once the representation of the ‘local community’ and its ‘environment’ have been established (or secured) through sustained articulations of equivalences of responsible engagements with locals and implementations of global standards and certifications, these representations can travel beyond the local sphere through stories in corporate reports and media communication. This part of the ontological politics of CSR is best described by Latour’s (1991) concept of ‘immutable mobiles,’ which refers to an entity, or object, that can travel from one place to the other without suffering from distortion, loss or corruption. For example, a map is an immutable mobile that makes it possible to bring the remote land back to the center while not taking the actual piece of land with it. Latour (1991) shows how much energy needs to be spent and how much technology has to be put in place to sustain an immutable mobile (e.g., measure the exact geographical position of an island, produce data related to the location of an oilfield, or create the statistics that go into an economic projection).

The representations of the community produced in CSR reports and communications are a type of immutable mobile similar to that of a map, enabling a certain kind of representation of ‘the community’ to travel across space and time to the corporate head office and its key stakeholders (e.g., investors, customers, suppliers, and citizens in the country where the head office is based). All CSR practices explained above (investing in local development projects, setting standards, engaging in dialogues) involve a vast amount of energy and technologies that sustain the immutability of the singularized local world. These singularized representations of ‘the community’ can then be spread across the world through corporate (and media) texts, images, and videos without being altered along the way, ‘crowding out’ other images that risk causing ‘ontological fractures’ (Sepúlveda, 2016) in how externals perceive local ways of worlding. These CSR representations also blend with other objects, such as financial instruments (e.g., green bonds), that create the appearance of shifting capital to more sustainable economic activity (see Maltais & Nykvist, 2020), or eco-labeled consumer goods (e.g., FSC-certified paper), creating the perception that the consumption of these products is beneficial for both the people and the planet.

These representations in corporate CSR reports and media communication will always be partial, as a lot of relevant information is not disclosed (Michelon et al., 2015; Parguel et al., 2011). When CSR is performed as singularized representation, it produces particular networks of human–nonhuman entanglements that make certain entities visible while obscuring others, which then ‘restricts the plurality of interpretations’ (Gond & Nyberg, 2017, p. 1137) of particular contested topics (e.g., climate footprints, sustainability). Gond and Nyberg (2017) assert that, while CSR representations are continuously produced and upheld through entangled actors and relationships, they can come to be reassembled by the mobilizing forces of actors that reorient the representations of CSR. While this may be possible, Gond and Nyberg’s (2017) study does not account for the existence of a pluriverse of nonmodern ways of worlding. Due to the interpretational dominance of the ‘scientific’ and ‘objective’ modern representations of ‘the world’ (in singular), other local ways of being are portrayed as ‘cultural traits’ rather than different worlds, whose own ways of worlding and human–nonhuman relations are endangered by the presence of the corporation and its associated (extractive and CSR) practices. Thus, these worlds will never be able to be translated into such modern representations without losing parts of their own place-based particularities. It is, in fact, an impossibility for them to become part of the CSR agencements based on resource extraction practices for global markets, as that would require the dissolution of their own existences and their immersion into the OWW of the modern myth.

Our account of the political ontology of CSR shows a much more complicated picture of the world-making effects of these reporting devices. Viewing CSR as a world-making practice shows that we are not dealing with a partial account of the complex reality ‘out there.’ Instead, CSR contributes to sustaining and reproducing a particular kind of corporate and modern world. Through the three mechanisms of singularization discussed in this chapter, these CSR practices obscure the pluriverse and its alternative world-making futures in place. Through concrete engagements with local communities and their lived worlds, CSR both creates concrete stakes and stakeholders and converts a complex set of human–nonhuman relations into fixed representations of the environment, expressed through measurable units and indicators. These representations are thus aligned with the modern myth, and, through the continuous replication of CSR practices, the singularized reality of the OWW expands its occupation into novel territories and places.

## Discussion and Conclusion

In this article, we have examined CSR as an ontological practice that is directly tied to modernity, or what Law (2015) and Escobar (2016) have called OWW. We have argued that the PO of CSR plays an important role in shaping place in ways that reproduce modern ontological assumptions and legitimize extractive land-use practices. This extension of the ontological occupation of the OWW creates specific places and worlds, obscuring other ontologies, alternatives, and non-corporate ways of being in place, making radically different futures unthinkable (Escobar, 2020).

The purpose of this article has not been to discuss whether the lives of locals are improved by CSR engagements or not. From within the modern vantage point, it might well be that the lives of many impoverished communities are improved by CSR practices. Having said that, due to the dire socio-ecological consequences that the OWW brings with it, such ‘half-truths’ are increasingly being scrutinized, not just by PO enthusiasts or postcolonial scholars, but by a wide variety of communities, social movements, activists, and researchers from different disciplinary backgrounds (Heikkurinen et al., 2016; Hickel, 2020; Parker, 2018). The purpose of our article has also not been to repeat those critical assessments of CSR that highlight its irresponsibility, greenwashing, imperialism, and (neo)colonial violence. Such CSR accounts are already well established among critical and postcolonial scholars (e.g., Alcadipani & de Oliveira Medeiros, 2020; Banerjee, 2008, 2018; Blowfield & Frynas, 2005; Fleming & Jones, 2012; Hanlon & Fleming, 2009; Maher, Huenteao, et al., 2021; Mena et al., 2016; Özkazanç-Pan, 2018; Rhodes & Fleming, 2020). Instead, we have argued that, through its enactment, CSR makes a particular ontological reality come into being, obscuring, and threatening the existence of other place-based worlds. Our article hence makes three interlinked contributions to the debate on the politics of CSR.

First, our analysis of the political ontology of CSR shows that the political struggle works through singularization and the streamlining of the complexities of place into one particular way of being and relating to the land. This stands in stark contrast to the processual and micro-foundational perspectives of the politics of CSR (Gond & Moser, 2021; Gond et al., 2017), which suggest that political struggles involve a plurality of logics, shaping the material outcomes of CSR (Demers & Gond, 2020; Gond & Nyberg, 2020; Jensen & Sandström, 2020). From a political ontological frame, we see that there is one dominant logic—the modern—that plays an important role in the ontological struggles of place-making. Thus, instead of involving a plurality of discourses, institutions and practices that change over time (Acosta et al., 2021), the political ontology of CSR works by reproducing the modern myth through different institutional arrangements, such as corporate philanthropy, multi-stakeholder dialogues, and global environmental standards and indicators. Thus, with the help of modernity’s dominance over other ways of worlding and the definition over what is un/real, CSR, in its current form, sustains the OWW and the idea of a singular environment ‘out there.’ By occluding all other possible (non-extractive) ways of performing the world in place, CSR effectively sets the political limits of what can exist and what can be imagined in the networks assembled by corporations and their stakeholders.

Second, the understanding of the dynamics of political struggles over how corporations should be governed and regulated (Djelic & Etchanchu, 2017; Mäkinen & Kasanen, 2016; Mäkinen & Kourula, 2012; Sorsa & Fougère, 2020) completely changes if stakes (e.g., the corporate funded hospitals, schools, sports grounds) are continuously crafted through the world-making practices of CSR. Thus, through the creation of stakeholders and particular kinds of environmental indicators, CSR streamlines, subordinates and eliminates the multiplicity of other ways of worlding, stabilizing the things at stake and settling the ontological struggles over what is not only right and wrong but what is real and unreal as well as imaginable and unimaginable. These stakes and environments are not crafted according to a particular organizational entity’s (e.g., government, corporation, NGOs) political interests or values but are defined by how modernity reproduces the OWW in accordance with its ontological assumptions of human–nature separation, linearity of time, and the colonial difference. ‘Sustainability’ and ‘responsibility’ are hence terms that are based on standardized templates negotiated in corporate-dominated global governance systems in which locals cannot raise their voices on their own terms. This has concrete consequences for the kinds of realities that are materialized, both in the place of local communities and the global space of CSR reporting. Thus, the question to be addressed from a PO perspective is not so much whether corporations or governments should be in charge of regulating corporate actions (Rhodes and Fleming, 2019). Rather, PO asks what kinds of realities and worlds are allowed to exist when decisions about the future of place are made.

Third, critical and postcolonial perspectives tend to view CSR as mostly greenwashing or propaganda (Mahoney, 2013; Siano et al., 2017; Lee et al., 2018; Hanlon & Fleming, 2009), hiding the violence used to tame local opposition groups (Banerjee, 2000, 2008, 2018; Banerjee et al., 2021). As discussed above, the PO approach is very aware of the violence involved in creating and reproducing the OWW. As such, CSR needs to be understood as a regime of violence (Böhm & Pascucci, 2020). Yet, then PO proceeds by offering a different analysis of the more subtle processes involved in the encounters between corporate and local actors. The promise that CSR brings to the local community in terms of ‘shared value creation’ (Porter & Kramer, 2011) acts as a ‘Trojan horse’ that subconsciously, and supported by the storyline of the modern myth, lure community members into the sphere of influence of the corporation (Infante, 2012). Similar to Whelan’s (2019) proposition that corporations create specific realities through what they produce and sell, so do they shape the worlds of those whose everyday lives become entangled with the corporate world through CSR. Here, the way CSR uses and diffuses specific words and stories really matters in material terms. This is not always an act of greenwashing, which would be the case if stories are produced without any anchoring in the lived experiences in place. Instead, PO alerts us to the process of how CSR stories produce particular kinds of worlds through the dialogues and material investments performed in the community, contributing, in a very tangible way, to the realities that they narrate in place. That is, instead of speaking of the relations between people and nonhumans that get interrupted or broken by extractive practices, these CSR stories focus their attention on the education centers, hospitals and housing projects that are built, and the water quality and carbon emissions that are measured and labeled as responsible and sustainable. Whether these CSR practices are centered on the creation of shared values or the measurement of environmental indicators, they always redirect the attention away from the ontological conflicts that surround the use of land for extractive purposes and the violence involved in the destruction of locals’ relations to the web of life.

We are well aware that this article might have left a number of questions unanswered. We invite researchers to further explore the PO approach to understanding CSR. Let us hence highlight three potential avenues for future research.

First, we suggest that scholars interested in the politics of CSR should focus more on the subtle processes through which the presence of the corporations and their CSR initiatives legitimize dispossessions and transform ontologies and places. How is CSR used to convert community members into stakeholders? How do human–nonhuman relations change with corporate presence on the land (e.g., open pit mining, large-scale fishing, monocultural tree plantations). Such detailed analyses would explain the ontological struggles over the things at stake in these types of land conflicts, where land (and water and nature) is not a singular entity but multiple, embodied by the practices that sustain different realities and worlds. Relatedly, PO invites scholars to understand sustainability not just as a representation in various CSR reporting devices (Gond & Nyberg, 2017) but as practiced in place. Hence, there is a need to understand sustainability as a relational construct (Ergene et al., 2021). This requires that we as scholars detach our own thinking from the modern ontology in terms of how we define the environment based on various standardized indicators that represent a more or less accurate reality out there. Instead, sustainabilities as practiced in place are always emergent and dependent on different organizational configurations and ways of worlding in different contexts. To account for the ontological multiplicity, it is important to point out that these sustainabilities are not just ‘earth centric’ (Banerjee & Arjaliès, 2021) but pluriversal because how people relate to the earth depends on the ontology that shapes their own world. Hence, pluriversal responsibilities are all those different ways of human and nonhuman worlding that respond to the larger web of life in a place-based community.

Second, the PO approach has relevance in many other domains than CSR. For example, future studies should pay more attention to the ontological dimension of the politics of responsibility and sustainability in global climate and biodiversity conservation negotiations. ‘Net-zero,’ ‘carbon offsetting’ and ‘nature-based solutions’ are all examples of the modern ontology in action, as states and corporations attempt to ‘green’ themselves. Most of these discourses and material practices either ignore, exclude, or obscure the pluriverse of other ontologies and worlds. What we need is a new sensibility toward place; a deep engagement with the ontological realities of specific territories and their human–nonhuman entanglements. At the same time, however, we also need to understand in more depth how experts, tools, and techniques—and other human–nonhuman actants—put such OWW tools in place to create not just legitimizing discourses but concrete stakes, stakeholders and environments that serve global circuits of ‘responsible’ and ‘sustainable’ trade and investment. As planetary crises such as biodiversity loss and climate change intensify, we will see a plethora of new ‘nature-based’ solutions that will put increasing pressure on the land. More and more land will be needed by the OWW for planting trees, carbon offsetting, growing food and biofuels, but also create rewilding zones. This will inevitably lead to an intensification of ontological conflicts between the OWW and other ontologies in the pluriverse. The PO approach sensitizes scholars to such ontological struggles.

Third, and perhaps most importantly, we suggest that there is an urgent need to see the pluriverse in action, directly engaging with other ontologies and world-making practices. Today, many movements threatened by corporate interventions on their lands do not only engage in anti-corporate resistance (Banerjee, 2018; Kraemer et al., 2013; Misoczky & Böhm, 2015; Pal, 2016), they also strategically engage in building alternatives to extractivist projects by drawing on their own (de)colonial histories and life-sustaining webs of relation in place (Ehrnström-Fuentes, 2022b; Nirmal & Rocheleau, 2019). The political projects of these movements combine ‘resistance with recovery, renewal, and/or reinvention of various interspecies and social relations in and across places and times (including connections with land and water)’ (Nirmal & Rocheleau, 2019, p. 472). These movements are not entangled with (ir)responsible corporate practices and worlds, rather they strategically engage in ‘reweaving worlds and restoring relations broken or threatened by capitalist/colonial interventions’ (ibid., p. 473). This involves a radical shift from anthropocentric views of organizations toward understanding organizing in relational, ontological and ecocentric ways (Ergene et al. 2021; Banerjee & Arjaliès, 2021; Heikkurinen et al., 2016). As such, PO alerts us to the need of relational (that is, ecocentric) and pluriversal approaches, helping researchers to take account of the ontological conflicts in action as different worlds meet and mingle. Thus, what PO adds to the debate on ‘relational ontologies’ is a way of thinking and analytical tools that help us study how the ontological politics ‘in-between worlds’ play out in different contexts as the modern/anthropocentric world loses its grip. This has to involve a deep understanding of how people relate to place and territory in their everyday practices, precisely because a world cannot exist without place. People live in a place; economies and livelihoods exist in places; ecologies are place-based and dependent on how people connect with the flow of the planetary spheres. As we have discussed, for many land-based communities place is not only a series of OWW-produced buildings, parks, streets, or even agricultural fields. Instead, place is a complex entanglement of human and nonhuman dimensions that communities are defending as their way of life. Here, it is important to make clear that the ontological politics of the pluriverse can manifest itself anywhere where grassroots movements through convivial life-making practices seek to overcome the singularized OWW path (Escobar, 2020; Kothari et al., 2019). That is, the pluriverse is by no means only tied to Indigenous communities and their ontologies but encompasses different types of transition initiatives, place-based collectives, grassroots movements that, through their practices, break with the universalizing assumptions of the modern world, while being committed to restoring and repairing their relations with the Earth (Escobar, 2020).

The task for us scholars who do critical work on the politics of CSR, is to take other worlds seriously, not consider them as utopian or relics of the past, but to seriously engage with the propositions they have to offer. Other economic horizons depend not only on discursive struggles among powerful actors, but on how we collectively reframe and re-articulate the story of place itself (Gibson-Graham, 2007). It is in the borderlands between corporate and alternative worlds where the politics over the future of the pluriverse takes place. This involves conflicts and struggles. To create alternative futures, communities will need to ‘break free’ through processes of decolonization that makes it possible to imagine different futures (Escobar, 2020). Such processes of decolonization tend to occur outside the field of influence of corporations and their CSR approaches (Escobar 2016, 2020; Nirmal & Rocheleau, 2019).
